# In Vivo Diuretic Activity of Hydromethanolic Extract and Solvent Fractions of the Root Bark of *Clerodendrum myricoides* Hochst. (Lamiaceae)

**DOI:** 10.1155/2020/1718708

**Published:** 2020-12-10

**Authors:** Gebrelibanos Gebremichael Welu, Ebrahim M. Yimer, Haftom Gebregergs Hailu, Dayananda Bhoumik, Mehari Meles Lema

**Affiliations:** ^1^Department of Pharmacology and Toxicology, College of Health Sciences, Mekelle University, Mekelle, Ethiopia; ^2^Pharmacy and Toxicology Unit, Department of Pharmacy, College of Health Sciences, Aksum University, Aksum, Ethiopia; ^3^Department of Pharmacy, College of Medicine and Health Sciences, Wollo University, Dessie, Ethiopia; ^4^Ethiopian Public Health Institute, Addis Ababa, Ethiopia

## Abstract

**Introduction:**

*Clerodendrum myricoides* (Lamiaceae) has been traditionally used for the treatment of various ailments, including body swelling and urine retention. The present study aimed to evaluate the diuretic activity of a crude extract and solvent fractions of the root bark of *C. myricoides*. *Methodology*. The coarsely powdered root bark of *C. myricoides* was extracted by a cold maceration method using 80% methanol. A portion of the extract was fractionated based on the polarity index of solvents to obtain chloroform, ethyl acetate, and aqueous fractions. To investigate the diuretic activity of the plant, rats were divided into fifteen groups. The normal control groups received either water or 2% tween 80, the standard group received furosemide (10 mg/kg), and the test groups were administered the hydromethanolic extract and solvent fractions at the doses of 100, 200, and 400 mg/kg by the oral route. The urine volume, urine pH, urine, and serum electrolytes were determined and compared with the standard and normal control groups.

**Results:**

The crude hydromethanolic extract, ethyl acetate, and chloroform fractions induced significant diuresis at a dose of 400 mg/kg (*P* < 0.001) compared to the aqueous fraction. The hydromethanolic extract at 200 mg/kg and 400 mg/kg also caused noticeable diuresis (*P* < 0.001) compared to the standard, furosemide. Rats treated with hydromethanolic extract, ethyl acetate, and chloroform fractions showed delayed onset and prolonged diuresis in a dose-dependent fashion compared to the aqueous fraction (*P* < 0.05). The hydromethanolic extract and solvent fractions produced the highest saliuretic and natriuretic index compared to the standard, furosemide. The crude hydromethanolic extract also failed to produce any sign of toxicity up to 2000 mg/kg.

**Conclusion:**

From this study, the hydromethanolic extract and ethyl acetate fraction of the root bark of *C. myricoides* produced a prominent diuretic effect in rats.

## 1. Introduction

Substances that elevate the rate of urine flow and salt loss are known as diuretics [1, 2]. The net excretory effect of diuretic agents causes changes in urine flow, pH, and ionic compositions of urine and blood [3]. Diuretic agents are important to promote a net loss of excessive accumulated body fluids, salts, toxemias, and other accumulated metabolic products including urea [4]. However, the currently available diuretic agents are associated with numerous side effects and diuretic resistance in some patients. For instance, the recent global cohort studies indicated that the prevalence of diuretic resistance was estimated to be 20–35% in heart failure cases [5, 6].

Therefore, there is a need to look for alternative diuretics with a novel mode of action, better efficacy, and tolerable side effects profile. Medicinal plants are considered as a vital source for the development of potential therapeutic effective drugs. *C. myricoides* (Lamiaceae) is locally called “Surbetri or Shiwha” in Tigrigna [7, 8]. Most of the plant species under this genus are a rich source of biologically active secondary metabolites such as terpenes, tannins, steroids, phenolic acids, glycosides, flavonoids, and alkaloids [8]. A number of species from this genus were previously reported to have several pharmacological activities including diuretics action and antihypertensive [9, 10].

The methanolic leaf and root extracts of *C. myricoides* have been examined for different pharmacological activities. For instance, the leaf part was examined for the antiplasmodial [11], antifungal, and antibacterial activities [12]. Notable antimicrobial activity against all tested pathogens was also reported from the root part of this plant [13]. Besides, phytochemical screening of the methanolic root extract of *C. myricoides* showed the presence of flavonoids, terpenoids, phenols, glycosides, tannins, and saponins [14].

Based on the information obtained from the local community (Adwa, Tigray) and supported by ethnobotanical reports, the root bark of *C. myricoides* is claimed to promote diuresis. The powdered root bark of *C. myricoides* with the addition of water is orally administered for swelling in the body, urination problem, and/or urinary retention [8, 15].

The widespread use of the medicinal plant by local people entails the necessity of testing of their efficacy and safety profile [16]. Therefore, this study aimed to investigate the effect of both the hydromethanolic extract and different solvent fractions of the root bark of *C. myricoides* on the urine output, urine pH, and both urine and serum electrolytes of rats.

## 2. Materials and Methods

### 2.1. Drugs, Chemicals, and Reagents

Ketamine hydrochloride (Neon Laboratories Limited, India), furosemide (Changzhou Yabang Pharmaceutical, China), absolute methanol (Alpha Chemika, India), chloroform (Nice Chemicals, India), ethyl acetate (Fine Chem, India), tween 80 (Atlas Chemicals, India), and normal saline (Addis Pharmaceutical Factory, Ethiopia). All the other chemicals used were also laboratory and/or analytical grade.

### 2.2. Experimental Plant

The fresh root of *C. myricoides* was collected in December 2018 and identified, and specimens (voucher number of ETH/05/2011/2019) were deposited at the National Herbarium, Addis Ababa University. The roots were thoroughly washed with tap water; root bark was separated and cut to smaller sizes, dried under shade for two weeks, and coarsely powdered using a mechanical grinder.

### 2.3. Preparation of Crude Extract and Solvent Fractions

After drying, 1500 g of the coarsely powdered root bark of *C. myricoides* was macerated in 7.5 liters of 80% of methanol for 72 hr with occasional agitation using the orbital shaker. The macerate was separated using a triple-layered muslin cloth followed by Whatman No. 1 filter paper. The marc was resoaked twice to obtain sufficient yield following the same procedure. The combined filtrate was concentrated in the oven dryer at 40°C.

Then, 130 g of the hydromethanolic extract (HME) was allowed to suspend in 200 mL of distilled water. A 200 mL of chloroform was added into aqueous suspension and vigorously shaken in a separatory funnel and kept until a clear layer appeared. The bottom layer of the chloroform fraction was separated into a glass beaker. The aqueous residue was reshaken twice with the addition of the same volume of fresh chloroform following a similar protocol. The remaining aqueous residue was then mixed and shaken thrice with 200 mL of ethyl acetate following the above procedure (but the upper layer was ethyl acetate fraction). At the end, all the fractions were concentrated in the oven dryer at 40°C.

### 2.4. Experimental Animals

Swiss albino mice and Sprague–Dawley rats were obtained from the animal breeding house of the Department of Pharmacology and Toxicology, Mekelle University. Before starting the experiments, all the animals were housed individually in the standard plastic cage inside the experimental laboratory room (natural light/dark cycles) with free access to standard food and tap water. The study clearance was obtained from the Health Research Ethics Review Committee of the College of Health Sciences, Mekelle University, with protocol number 1538/2018.

### 2.5. Acute Oral Toxicity Study

The study was carried out by a limit test of the Organization for Economic Cooperation and Development Guideline No. 425 [17]. Five none pregnant female mice weighed between 25 g and 30 g and aged 8–12 weeks were used for this study. All mice fasted for food, but not for water, 4 hr before dosing and for 2 hr after administration of the extract. The dose was calculated according to the bodyweight of the fasted mice. Initially, one mouse was treated with a single dose of the extract, 2000 mg/kg. After a day, since the mouse survived, continued to administer a single dose (2000 mg/kg) for 4 mice. After administration of the extract, the mice were carefully observed for short-term toxicity profile up to 24 hr and up to 14 days for the mortality profile.

### 2.6. Grouping and Dosing

Either sex of Sprague–Dawley rats having a weight range of 200–250 g and age of 12–16 weeks were distributed into fifteen groups randomly (*n* = 6/group). The groups were randomly assigned as two normal control groups, one standard group and the rest (group IV up to XV) as *C. myricoides* treatment groups. Before commencing the experimentation, each rat was placed daily for 3 hr for a total of 3 days in the metabolic cage, for acclimatization [18]. The hydromethanolic extract and its AF were freshly prepared in distilled water, while the EAF and CF were dissolved in 2% tween 80 on the day of the experiment and administered orally. Accordingly, the first normal control group was treated with distilled water, and the second normal control group was treated with 2% tween 80. The standard group was treated with furosemide 10 mg/kg [19], while groups IV–XV were treated with the three different test doses (100, 200, and 400 mg/kg) of the root bark of *C. myricoides* HME and its solvent fractions.

### 2.7. Diuretic Activity

The screening of diuretic activity was performed using the model described by Lipschitz [20] and Kau [21]. The overnight fasted rats for food but not for water were hydrated using a single oral dose of normal saline 25 mL/kg [22]. At the beginning of the treatment, the fasted but hydrated rat's urinary bladder was emptied by gently compressing at the pelvic area and using the pulldown of their tails [23]. Then, the rats were administered the test doses as described in the section of grouping and dosing. Immediately after administration, the rats were placed individually in the metabolic cage. The excreted urine volume was collected and measured at the end of 1st hr, 2nd hr, 4th hr, 6th hr, and at the last 24th hr intervals after dosing [24]. Urine pH was measured from a fresh urine sample of each rat using a digital pH meter [23]. The collected urine was stored in a refrigerator (−80°C) for further urine analysis.

The percentage of urine excretion (formula (1)), diuretic action (formula (2)), and diuretic index (formula (3)) was calculated for all groups using the mean urine output at 6th and 24th hr. The obtained diuretic index was considered good if the resulted values were >1.5, moderate if the resulted values depict between 1.00 and 1.5, least if the resulted values range between 0.72 and 0.99, and nil if the resulted values < 0.72 [19, 23].

(1) $$\begin{array}{c} \text{Urinary excretion}=\frac{\text{Total urinary output}}{\text{Total volume of liquid administered}}*100, \end{array}$$

(2) $$\begin{array}{c} \text{Diuretic action}=\frac{\text{Urinary excretion of the test group}}{\text{Urinary excretion of the control group}}\text{,} \end{array}$$

(3) $$\begin{array}{c} \text{Diuretic index}=\frac{\text{Diuretic action of the test group}}{\text{Diuretic action of the standard group}}. \end{array}$$

### 2.8. Biochemical Analysis

At the end of the experiment, all rats were anesthetized using ketamine hydrochloride (75 mg/kg IP), and blood was collected from each rat through the retroorbital sinus [25]. The serum was separated by centrifugation at 2500 rotation per minute for 10 minutes for further serum electrolyte analysis. The urinary (from the 24 hr urine) and serum electrolyte contents including Na^+^, K^+^, and Cl^−^ were analyzed using Cobas^®^ 6000 analyzer series (Roche, Germany).

Using the data obtained from urine electrolytes analysis, saliuretic index was calculated for an individual Na^+^, K^+^, and Cl^−^ as a ratio of their concentrations in the treated groups compared to the normal control group (formula (4)) [26].

(4) $$\begin{array}{c} \text{Saliuretic index}=\frac{\text{Urinary }{\text{Na}}^{+},{\text{K}}^{+},{\text{Cl}}^{-}\text{ level in the test group }}{\text{Urinary }{\text{Na}}^{+},{\text{K}}^{+},{\text{Cl}}^{-}\text{ level in the control group}}. \end{array}$$

Using the data obtained from urine electrolytes analysis in the same group, natriuretic index or aldosterone secretion index (Na^+^ ratio to K^+^) (formula (5)) and carbonic anhydrase inhibition (CAI) index (Cl^−^ ratio to sum of Na^+^ + K^+^) (formula (6)) were calculated [26–28].

(5) $$\begin{array}{c} \text{Natriuretic index}=\frac{\text{Urinary }{\text{Na}}^{+}\text{ level in the same test group}}{\text{Urinary }{\text{K}}^{+\;}\text{ level in the same test group}}, \end{array}$$

(6) $$\begin{array}{c} \text{CAI index}=\frac{\text{Urinary }{\text{Cl}}^{-}\text{ level in the same test group}}{\text{Sum of urinary }{\text{Na}}^{+}+{\text{K}}^{+}\text{ level in the same test group}}. \end{array}$$

If the obtained Na^+^/K^+^ ratio >1, it indicates a satisfactory natriuretic index [29], and if > 2, it indicates favorable Na^+^ urinary excretion without excessive urinary K^+^ loss, but if > 10, it indicates a favorable K^+^ sparing effect [30]. If the calculated value of the Cl^−^/Na^+^+K^+^ ratio is between 0.8 and 1.00, it excludes CAI activities, but if below 0.8, it is considered to have a strong CAI index [31].

### 2.9. Data Analysis

The data in this study were analyzed using a statistical package for social science, version 20. The results of the study were expressed as mean ± standard error of the mean (SEM). Statistical significant differences were determined by one-way ANOVA followed by the post hoc Tukey test to compare urine volume, urine, and serum electrolyte concentration among the controls, standards, HME, and solvent fractions treated groups. *P* value < 0.05 was considered statistically significant.

## 3. Results

### 3.1. Percentage Yield of the Plant Extract

The percentage yield of HME was 35.62%, and among its solvent fractions, the AF had the highest percentage yield, 92.88%, while the CF, 6.33%, and the EAF, 0.75%, showed lower extraction yields.

### 3.2. Acute Oral Toxicity Study

The crude extract (HME) of the root bark of *C. myricoides* (at a dose of 2000 mg/kg) did not produce any physically visible sign of toxicity, up to 14 days of follow-up.

### 3.3. Diuretic Activity: Effect on Urine Output

**Figure 1:**
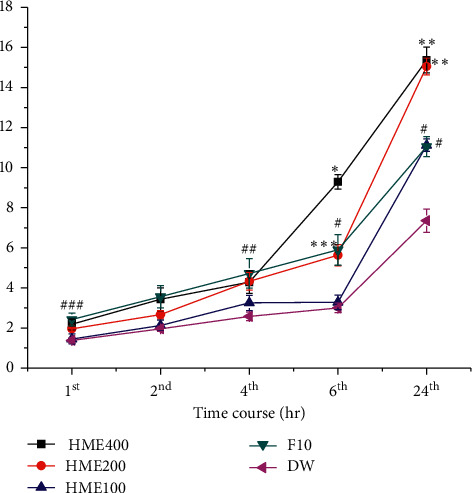
The effect of the hydromethanolic extract of the root bark of *Clerodendrum myricoides* on 24 hr urine volume in rats. Compared to HME: hydromethanolic extract (^a^400, ^b^200, ^c^100 mg/kg); ^d^compared to F10: furosemide 10 mg/kg; ^e^compared to DW: distilled water. ^1^*P* < 0.05; ^2^*P* < 0.01; ^3^*P* < 0.001. Key:  ^*∗*^(b3, c3, d3, e3),  ^*∗∗*^(c3, d3, e3), and  ^*∗∗∗*^(c1, e2) and ^#^(e3), ^##^(e2), and ^###^(e1).

The effect of HME on urine output is presented in Figure 1. All test doses of HME caused continual urine output until the 24th hr compared to the normal control group (*P* < 0.001). Both 200 mg/kg (*P* < 0.01) and 400 mg/kg (*P* < 0.001) test doses of HME produced better urine output starting from the 6th hr compared to the normal control group. The HME at 400 mg/kg also showed better diuresis starting from the 6th hr and continued until the 24th hr compared to furosemide (F10 mg/kg) treated rats (*P* < 0.001). The 200 mg/kg dose also caused an increment of urine output at the last 24th hr compared to F10 mg/kg treated rats (*P* < 0.001).

Unlike the HME, all the test doses of AF failed to show a significant increment of urine output (Table 1). The EAF exhibited increment of urine output at 400 mg/kg starting from the 6th hr compared to the normal control group (*P* < 0.05). It also (400 mg/kg) displayed a noticeable and continuous increment of urine output until the 24th hr compared to the normal control group (*P* < 0.001). In this fraction, both the 200 mg/kg and 100 mg/kg also caused a significant increment of urine output compared to the normal control at the last 24th hr. The CF of the HME produced a significant increment of urine output in a dose-dependent manner compared to the normal control group at the 24th hr.

**Figure 2:**
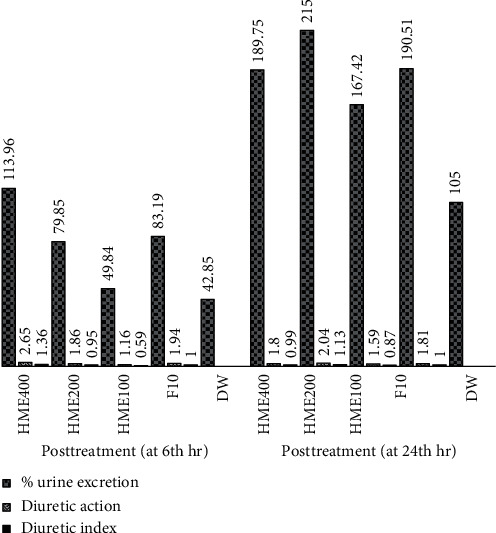
Diuretic index of the hydromethanolic extract of the root bark of *Clerodendrum myricoides* at 6th hr and 24th hr. HME, hydromethanolic extract; F10, furosemide 10 mg/kg; DW, distilled water.

Moreover, the highest and lowest percentages of urinary excretion, diuretic action, and diuretic index were observed at 400 mg/kg and 100 mg/kg doses of the HME, respectively (Figure 2). However, at 24th hr, the highest percentage of urinary excretion, diuretic action, and diuretic index at a dose of 200 mg/kg of the HME with values of 215%, 2.04, and 1.13, respectively, was observed.

Furthermore, the highest percentage of urinary excretion, diuretic action, and diuretic index was also observed at the largest test dose of EAF and CF at both 6th hr and 24th hr (Table 2). Unlike the HME, EAF, and CF, all the test doses of the AF appeared to have “nil” (<0.72) diuretic index.

### 3.4. Urine pH

**Figure 3:**
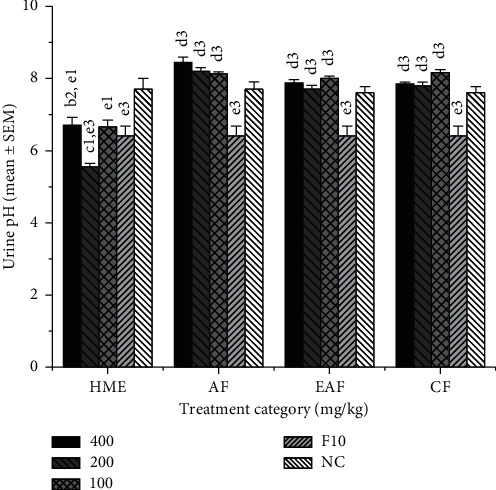
Urine pH of rats (*n* = 6) treated with the hydromethanolic extract and solvent fractions of the root bark of *Clerodendrum myricoides.* HME, hydromethanolic extract (^a^400, ^b^200, ^c^100 mg/kg); AF, aqueous fraction (^a^400, ^b^200, ^c^100 mg/kg); EAF, ethyl acetate fraction (^a^400, ^b^200, ^c^100 mg/kg); CF, chloroform fraction (^a^400, ^b^200, ^c^100 mg/kg); ^d^F10, furosemide 10 mg/kg; ^e^NC, normal control (either distilled water compared to HME and AF or 2% Tween 80 compared to EAF and CF). ^1^*P* < 0.05; ^2^*P* < 0.01; ^3^*P* < 0.001.

As displayed in Figure 3, both the collected urine from the groups treated with HME and F10 mg/kg treated rats were found to have slightly lower urine pH while the test doses of the AF, EAF, and CF of the HME have caused slightly alkaline urine pH.

### 3.5. Electrolyte Content of the Urine

The effect of HME and solvent fractions of the root bark of *C. myricoides* on urinary electrolyte excretion is shown in Table 3. The HME at 200 mg/kg and F10 mg/kg treated rats has produced the highest urinary K^+^ excretion (*P* < 0.01). Besides, the 200 mg/kg of the HME showed an increment of urinary Cl^−^ excretion compared to the normal control group and F10 mg/kg treated rats (*P* < 0.05). The least urinary Na^+^, K^+^, and Cl^−^ excretion was observed at 400 mg/kg of the HME of the plant. Compared to the test doses of the HME, the 200 mg/kg showed comparable saliuretic index with the F10 mg/kg treated rats. The least saliuretic index and the highest natriuretic index values were observed at 400 mg/kg compared to the F10 mg/kg treated rats. All the test doses of HME have produced <0.8 CAI index.

The AF at 400 mg/kg caused increment of K^+^ (*P* < 0.01) and Cl^−^ (*P* < 0.001) urinary excretion compared to the normal control group. The EAF at 200 mg/kg (*P* < 0.01) and 400 mg/kg (*P* < 0.05) showed substantial urinary Na^+^ excretion compared to the normal control group and F10 mg/kg treated rats. The CF at 400 mg/kg caused increment of urinary Na^+^, K^+^, and Cl^−^ excretion compared to the normal control group (*P* < 0.05), (*P* < 0.01), and (*P* < 0.001), respectively. Furthermore, the rats treated with larger test doses of AF, EAF, and CF were also found to have a higher saliuretic index than F10 mg/kg treated rats. In addition, rats treated with AF (at 100 and 200 mg/kg), EAF (at 200 and 400 mg/kg), and CF (at 100 mg/kg) showed higher natriuretic index (>1). The least CAI index was observed at both 200 mg/kg and 400 mg/kg doses of EAF and that of F10 mg/kg treated rats.

### 3.6. Electrolyte Content in the Serum

The effect of HME and solvent fractions of the root bark of *C. myricoides* on serum electrolyte levels is shown in Table 4. The rats treated with 400 mg/kg of HME appeared to have higher K^+^ serum level compared to that of F10 mg/kg treated rats (*P* < 0.01). Higher serum Na^+^ (*P* < 0.01) and Cl^−^ (*P* < 0.05) levels were observed at 100 mg/kg of the HME compared to the HME at 400 mg/kg. Substantial increment of the serum Na^+^ level was observed at 100 mg/kg (*P* < 0.01) and 200 mg/kg (*P* < 0.05) of AF compared to the normal control group. All test doses of AF, however, showed decrement of the serum K^+^ level compared to the normal control group (*P* < 0.001).

The rats treated with the 100 mg/kg (*P* < 0.001) of EAF were found to have higher serum Na^+^ level compared to the normal control. When compared all the test doses of EAF, it was observed that the 200 mg/kg caused higher serum K^+^ level compared to the 400 mg/kg (*P* < 0.01). The CF at 200 mg/kg and 400 mg/kg treated rats also showed a substantial serum Na^+^ level increment compared to the normal control and F10 mg/kg treated rats (*P* < 0.01). The minimal serum K^+^ level was observed at both doses of 100 mg/kg and 200 mg/kg compared to the normal control (*P* < 0.001).

## 4. Discussion

Diuretic agents are substances when introduced into a biological system, and they are increasing the net loss of urine and salts [30]. Urine and serum electrolyte, urine volume, and urine pH were measured as a parameter to evaluate the diuretic activity of the root bark of *C. myricoides* in rats used in this study.

The rats treated with three test doses of HME, EAF, and CF, especially at maximum test doses, showed a significant increment of urine output, starting from the 6th hr and continued till the 24th hr compared to the normal control. However, among the fractions, AF failed to reveal a significant increment of urine output. This could be attributed due to the existence of higher concentration of the diuretic active ingredient(s) in the HME and variation in type, quality, and quantity of active constituent(s) presented in these fractions [32, 33].

The delayed diuresis onset with the HME and its EAF and CF may be ascribed due to the slow absorption properties of the responsible active ingredient(s). The long diuresis duration of action with the HME and its EAF and CF throughout the study period (until the 24th hr) may be attributed due to the slow clearance properties of the responsible ingredient(s) [27, 28, 34].

The extracts showed different degrees of the percentage of urinary excretion, diuretic action, and diuretic index at both 6th hr and 24th hr. The higher percentage of urinary excretion, diuretic action, and the diuretic index was noticed from the HME followed by EAF and CF, while the lowest was noticed in the AF. The percentage of urinary excretion, diuretic action, and diuretic index of the HME, EAF, and CF showed to rise in a dose-dependent fashion.

Based on the estimated diuretic index, the diuretic potential of the HME and solvent fractions was ranked as ‘‘nil,” ‘‘least,” ‘‘moderate,” and ‘‘good,” if the calculated values were <0.72, 0.72 – 0.99, 1.00 – 1.5, and >1.5, respectively [23, 35]. Consequently, the HME (at 400 and 200 mg/kg) and EAF at 400 mg/kg showed a ‘‘moderate” diuretic index. The CF at 400 mg/kg has a ‘‘least” diuretic index, while the AF elicited a ‘‘nil” diuretic index. Therefore, this clearly showed that the ingredient(s) presented in the HME and EAF might be considered as the responsible constituent for the observed superior diuretic index.

The HME caused a significant increment in urine K^+^ and Cl^−^ excretion with lesser Na^+^ and Cl^−^ in serum, without serum K^+^ level alteration as compared to that of F10 mg/kg treated rats. The HME, especially at 200 mg/kg, was able to elicit saliuretic index as closer to F10 mg/kg treated rats. This could be related to the presence of higher K^+^ content in the plant extract and might have advantageous during hypokalemic conditions [19].

Loop diuretics enhance urinary outflow and excretion of Na^+^, K^+^, and Cl^−^ [36]. When loop diuretics inhibit Na^+^/K^+^/2Cl^−^ cotransporter at the thick ascending limb of the loop of Henle, it leads to increased Na^+^ load to the distal convoluted tubule, which indirectly causes urine acidification [36]. Hence, the HME caused significant urine K^+^ and Cl^−^ excretion (at 200 mg/kg), satisfactory natriuresis index (at 400 mg/kg), and significant acidic urine at all test doses. Therefore, it can be suggested that HME of the plant might have loop diuretic like the mechanism of action.

Besides, both the EAF and CF caused increment of urinary Na^+^, K^+^, and Cl^−^ excretion with significant alkalization of urine. As a result, the diuretic activity of the EAF and CF may be different from loop-like diuretics because loop diuretics cause marked natriuresis and urine acidification [16]. It is reasonable to suggest that the constituent(s) present in the root bark of *C. myricoides* seems to have different diuretic mechanisms of action.

The Na^+^/K^+^ ratio if > 1, 2, and 10 indicates satisfactory natriuresis, a favorable natriuresis, and favorable K^+^ sparing activity, respectively [29, 30]. Accordingly, the HME and EAF showed a satisfactory natriuretic index. However, in the present study, none of the test doses of HME and solvent fractions showed a favorable natriuretic index and K^+^ sparing activity. These findings are concurred with other previous diuretic plant studies (similar in the family), which have shown satisfactory natriuretic index and no K^+^ sparing activity [37].

The Cl^−^/Na^+^+K^+^ ratio in a test substance can predict the modes of the diuretic mechanism of the CAIs effect [38]. If the Cl^−^/Na^+^+K^+^ ratio is between 0.8 and 1.00, it excludes the CAI effect, but if below 0.8, it is considered to have strong CAI activity [31]. In this study, excluding the AF and CF, both the HME and EAF showed a strong CAI effect. This indicates that these fractions possibly have a different diuretic mode of action.

Collectively, the diuretic activity of the HME and its EAF was found to be highly potent when compared to the CF and AF. This indicates that the pharmacologically active ingredient(s) of the root bark of *C. myricoides* might have better solubility nature in the 80% methanol and better fractionated by ethyl acetate than other solvents used. Moreover, the results indicated that the diuretic activity of the HME is found to be higher than all fractions and comparable with a single oral dose (10 mg/kg) of furosemide. This might be due to the fact that the crude extract (HME) contains more pharmacologically active ingredient(s) and might act synergistically [39].

It was previously stated to contain flavonoids, terpenoids, phenols, glycosides, tannins, and saponins from the root part of the plant [14]. Diuretic activities of these detected constituents were studied in several previously conducted efficacies of plant-based studies, and their marked diuretic effects were reported [40–46]. As a result, the presence of the mentioned and other constituents might be coextracted components that could partly be responsible for the observed diuresis, natriuretic, and saliuretic activities of the root bark of *C. myricoides* [47, 48].

Regarding the safety profile of the plant, the HME and all solvent fractions of the experimental plant did not appear to have any physically visible signs and histological toxicity, as well as no death was recorded up to the dose of 2000 mg/kg of 14 days follow-up. Besides, no sign of toxicity and death was recorded during the diuretic activity study period.

## 5. Conclusion

The present study revealed that the rats treated with HME and EAF of the root bark of *C. myricoides* showed significantly delayed onset and dose-dependent prolonged diuresis, natriuretic effect, and CAIs effect. Both the medium and highest test doses of the HME showed remarkable diuresis. This could partly explain the traditionally claimed uses of *C. myricoides* as a diuretic agent. The CF produced lesser diuresis, while the AF devoid to show significant diuresis, but both showed the saliuretic effect at larger test dose. The HME and all of the solvent fractions were found to be safe up to 2000 mg/kg. However, further studies are required to isolate, purify, structurally elucidate, and propose the possible mechanisms of diuretic action and to assess the long-term efficacy as well as safety profile.

## Figures and Tables

**Table 1:** The effect of solvent fractions of the root bark of *Clerodendrum myricoides* on 24 hr urine volume in rats.

Dose (mg/kg)	^*∗*^Urine volume (mL)				
	1st hr	2nd hr	4th hr	6th hr	24th hr
AF400	0.33 ± 0.21^c2, d3, e1^	0.75 ± 0.47^d3^	1.83 ± 0.38^d3^	2.75 ± 0.35^d3^	8.33 ± 0.83^d1^
AF200	0.33 ± 0.21^c2, d3, e1^	1.66 ± 0.55^d1^	2.33 ± 0.16^d2^	3.66 ± 0.27^d2^	8.50 ± 0.21^d1^
AF100	1.50 ± 0.18^d1^	2.33 ± 0.10	2.83 ± 0.05^d1^	2.83 ± 0.52^d3^	7.83 ± 0.36^d2^
EAF400	1.05 ± 0.46	2.67 ± 0.42	4.33 ± 0.40	5.70 ± 0.31^e1^	11.54 ± 0.50^c1,e3^
EAF200	1.00 ± 0.44	2.15 ± 0.45	3.45 ± 0.31	4.45 ± 0.27	9.95 ± 0.34^e3^
EAF100	1.50 ± 0.18	3.61 ± 0.35	3.74 ± 0.33	4.62 ± 0.50	9.09 ± 0.62^e1^
CF400	1.58 ± 0.32	2.95 ± 0.20	3.91 ± 0.15	5.25 ± 0.21^e2^	10.86 ± 0.44^b1,e3^
CF200	2.25 ± 0.51	3.12 ± 0.17	2.25 ± 0.50^d2^	2.50 ± 0.54^d3^	10.50 ± 0.78^b1,e3^
CF100	1.50 ± 0.18	2.12 ± 0.12^d1^	2.91 ± 0.08^d1^	3.97 ± 0.20^d1^	8.12 ± 0.48^d2^
F10	2.42 ± 0.32^e1^	3.56 ± 0.55	4.72 ± 0.74^e2^	5.9 ± 0.76^e3^	11.05 ± 0.50^e3c^
DW	1.38 ± 0.14	1.96 ± 0.13	2.58 ± 0.21	3 ± 0.24	7.35 ± 0.58
2%TDW	1.37 ± 0.32	2.29 ± 0.40	2.83 ± 0.30	3.32 ± 0.24	6.66 ± 0.42

^*∗*^Results are expressed as mean ± SEM (*n* = 6). Compared to AF: aqueous fraction (^a^400, ^b^200, ^c^100 mg/kg); ^d^compared to F10: furosemide 10 mg/kg; ^e^compared to DW: distilled water; compared to EAF: ethyl acetate fraction (^a^400, ^b^200, ^c^100 mg/kg); compared to CF: chloroform fraction (^a^400, ^b^200, ^c^100 mg/kg); ^d^compared to F10: furosemide 10 mg/kg; ^e^compared to 2%TDW: 2% Tween 80. ^1^*P* < 0.05; ^2^*P* < 0.01; ^3^*P* < 0.001.

**Table 2:** Diuretic index of solvent fractions of the root bark of *Clerodendrum myricoides* at 6th hr and 24th hr.

Dose (mg/kg)	Posttreatment (at 6th hr)			Posttreatment (at 24th hr)		
	Urinary excretion	Diuretic action	Diuretic index	Urinary excretion	Diuretic action	Diuretic index
AF400	37.11	0.86	0.44	112.56	1.07	0.59
AF200	58.74	1.37	0.70	137.09	1.30	0.71
AF100	47.10	1.10	0.56	130.5	1.24	0.68
EAF400	95	1.59	0.94	192.33	1.73	1.01
EAF200	74.16	1.24	0.73	165.83	1.49	0.87
EAF100	76.87	1.29	0.76	151.5	1.36	0.79
CF400	84.67	1.42	0.84	175.16	1.57	0.91
CF200	40.91	0.68	0.40	172.13	1.55	0.90
CF100	63.16	1.06	0.63	135.33	1.21	0.70
F10	100.16	1.68	1	190.51	1.71	1
DW	42.85	—	—	105	—	—
2%TDW	59.46	—	—	111	—	—

AF, aqueous fraction; F10, furosemide 10 mg/kg; DW, distilled water; EAF, ethyl acetate fraction; CF, chloroform fraction; and 2%TDW, 2% Tween 80 in distilled water.

**Table 3:** The effect of the hydromethanolic extract and solvent fractions of the root bark of *Clerodendrum myricoides* on urine electrolyte in rats.

Dose (mg/kg)	^*∗*^Urine electrolyte (mmol/l)			Saliuretic index			Na^+^/K^+^	Cl^−^/Na^+^+K^+^
	Na^+^	K^+^	Cl^−^	Na^+^	K^+^	Cl^−^		
HME400	85.33 ± 8.30	66.38 ± 14.77^b2,d2^	106.83 ± 10.27^b3^	0.88	0.59	0.77	1.28	0.70
HME200	121.16 ± 7.60	124.18 ± 6.34	181.83 ± 7.52^c3,d1,e1^	1.26	1.11	1.31	0.97	0.74
HME100	87.66 ± 16.49	89.11 ± 7.44	114.66 ± 11.60	0.90	0.80	0.80	0.98	0.64
AF400	151.16 ± 15.09	192.50 ± 24.05^b2,c2,d1,e2^	369.66 ± 36.74^b3,c3,d3,e3^	1.56	1.73	2.67	0.78	1.07
AF200	116.33 ± 16.50	110.55 ± 13.85	164.33 ± 20.72	1.20	0.99	1.18	1.05	0.72
AF100	137.33 ± 15.72	102.66 ± 7.22	204.16 ± 14.85	1.42	0.92	1.47	1.33	0.85
EAF400	137.83 ± 4.84^d1,e1^	114.31 ± 8.75	172.50 ± 5.61	1.36	1.01	1.27	1.20	0.68
EAF200	146.83 ± 7.66^d2,e2^	110.53 ± 9.56	175.66 ± 8.72	1.45	0.98	1.29	1.33	0.68
EAF100	122.33 ± 7.51	126.45 ± 7.40	229.66 ± 27.97^d2,e1^	1.21	1.12	1.69	0.96	0.92
CF400	143.33 ± 8.38^b3,e1^	166.75 ± 7.74^b3,c2,e2^	287.00 ± 35.24^b3,d3,e3^	1.43	1.48	2.11	0.85	0.92
CF200	72.16 ± 13.77^c1^	74.40 ± 12.15^d2^	119.16 ± 23.22^c2^	0.71	0.66	0.87	0.96	0.81
CF100	119.66 ± 6.75	107.73 ± 6.23	242.33 ± 14.16^d1,e2^	1.18	0.96	1.78	1.11	1.06
F10	102.66 ± 13.13	129.58 ± 17.04	140.83 ± 11.74	1.01	1.15	1.03	0.79	0.60
DW	96.50 ± 0.67	110.88 ± 3.89	138.33 ± 3.89	—	—	—	0.87	0.67
2%TDW	100.83 ± 2.89	112.16 ± 4.33	135.66 ± 6.18	—	—	—	0.89	1.59

^*∗*^Results are expressed as mean ± SEM (*n* = 6). Compared to HME: hydromethanolic extract (^a^400, ^b^200, ^c^100 mg/kg); compared to AF: aqueous fraction (^a^400,^b^200,^c^100 mg/kg); ^d^compared to F10: furosemide 10 mg/kg; ^e^compared to DW: distilled water; compared to EAF: ethyl acetate fraction (^a^400, ^b^200, ^c^100 mg/kg); compared to CF: chloroform fraction (^a^400, ^b^200, ^c^100 mg/kg); ^d^compared to F10: furosemide 10 mg/kg; ^e^compared to 2%TDW: 2% Tween 80 in distilled water. ^1^*P* < 0.05; ^2^*P* < 0.01; ^3^*P* < 0.001. Saliuretic index = Na^+^, K^+^, Cl^−^ of the test group/Na^+^, K^+^, Cl^−^ of the control group; natriuretic index = Na^+^/K^+^ and CAI index = Cl^−^/Na^+^+K^+^ in the same group.

**Table 4:** The effect of the hydromethanolic extract and solvent fractions of the root bark of *Clerodendrum myricoides* on serum electrolyte in rats.

Dose (mg/kg)	^*∗*^Serum electrolyte (mmol/l)		
	Na^+^	K^+^	Cl^−^
HME400	135.00 ± 4.29^c2,d1,e1^	12.75 ± 3.19^b1,c2,d2^	93.50 ± 2.84^c3,d2,e2^
HME200	142.00 ± 1.63	5.41 ± 0.16	99.00 ± 1.29
HME100	147.66 ± 0.55	5.10 ± 0.07	103.16 ± 0.40
AF400	146.33 ± 0.98	4.46 ± 0.29^e3^	102.50 ± 0.76
AF200	147.83 ± 0.87^e1^	4.26 ± 0.07^e3^	102.66 ± 0.71
AF100	148.00 ± 0.63^e2^	3.96 ± 0.14^d1,e3^	104.33 ± 0.84
EAF400	148.16 ± 0.40	4.48 ± 0.18^e2^	101.00 ± 0.36
EAF200	145.00 ± 2.19	6.03 ± 0.58^c2^	104.00 ± 1.23
EAF100	150.83 ± 2.18^e1^	4.08 ± 0.08^e3^	104.33 ± 1.22
CF400	149.83 ± 1.51^d2,e2^	5.25 ± 0.47	102.83 ± 1.16
CF200	149.00 ± 0.85^d2,e1^	3.98 ± 0.11^e3^	100.66 ± 0.55
CF100	146.16 ± 0.47	4.03 ± 0.09^e3^	101.83 ± 0.16
F10	145.16 ± 0.79	5.18 ± 0.15	101.83 ± 0.30
DW	144.00 ± 0.36	7.06 ± 0.53	101.83 ± 0.47
2%TDW	144.33 ± 0.33	6.50 ± 0.55	102.16 ± 0.47

^*∗*^Results are expressed as mean ± SEM (*n* = 6). Compared to HME: hydromethanolic extract (^a^400, ^b^200, ^c^100 mg/kg); compared to AF: aqueous fraction (^a^400, ^b^200, ^c^100 mg/kg); ^d^compared to F10: furosemide 10 mg/kg; ^e^compared to DW: distilled water; compared to EAF: ethyl acetate fraction (^a^400 ,^b^200, ^c^100); compared to CF: chloroform fraction (^a^400, ^b^200, ^c^100 mg/kg); ^d^compared to F10: furosemide 10 mg/kg; ^e^compared to 2%TDW: 2% Tween 80 in distilled water. ^1^*P* < 0.05; ^2^*P* < 0.01; ^3^*P* < 0.001.

## Data Availability

The data used to support the findings of this study are available from the corresponding author upon request.
